# A Case of Resection of Multiple Dural Cavernous Hemangiomas Mimicking a Convexity Meningioma

**DOI:** 10.7759/cureus.110994

**Published:** 2026-06-16

**Authors:** Yusuke Hara, Keisuke Miura, Yoshifumi Okada

**Affiliations:** 1 Neurosurgery, Kitahara International Hospital, Kitahara Neurosurgical Institute (KNI), Hachioji, JPN

**Keywords:** angiography, cavernous hemangioma, convexity tumor, craniotomy tumorectomy, meningioma

## Abstract

The patient was a 55-year-old woman who presented with a chief complaint of a dull headache. An MRI scan revealed three tumors in the parietal and occipital regions, near the superior sagittal sinus. The patient was observed for suspected meningioma. After six years, a slight tumor enlargement was observed on follow-up imaging, and because the patient requested treatment, surgical resection was performed. Preoperative assessment revealed no developmental venous anomalies on MRI, no hemosiderosis on T2-weighted imaging, and a dural tail sign on contrast-enhanced T1-weighted imaging. However, angiography did not detect any feeding vessels. Postoperative pathological examination of the resected tumors diagnosed them as dural cavernous hemangiomas. Based on this case, we recommend performing angiography during the preoperative assessment when meningioma of the parasagittal region is the primary differential diagnosis and considering dural cavernous hemangioma as an alternative diagnosis.

## Introduction

Cavernous hemangioma (CH) has been observed in 0.4-0.5% of the population and has been documented as responsible for 5-13% of all vascular malformations of the central nervous system [1]. It most frequently occurs within the brain parenchyma, but rare cases of CH originating from the dura mater have also been reported. Dural cavernous hemangioma (DCH) originates from the dural sinuses, falx cerebri, falx cerebelli, skull base dura mater, or labyrinthine apex and apex region dura mater. The majority of these DCHs, also known as cavernous sinus CH, are typically found in the middle cranial fossa. The number of reported cases of apex DCH is less than 30 [2,3]. Reports of multiple DCHs arising in the tentorial region are, moreover, rare. While CHs within the brain parenchyma typically exhibit hemosiderin deposition within the lesion and at its margins on MRI scans, DCHs do not demonstrate the same MRI characteristics and may occasionally present with the dural tail sign. Consequently, these findings have the potential to result in an erroneous diagnosis of DCH as a meningioma [3]. In the present case, the patient underwent resection of two out of three tumor-like lesions near the superior sagittal sinus (SSS) in the parietal and occipital regions. The pathological diagnosis was DCH. Prior to the surgical intervention, the preoperative diagnosis was suspected to be a meningioma, as there was a presence of hemosiderin deposition within or at the margins of the lesion, and the dural tail sign was present. The final diagnosis was DCH. In light of the preoperative digital subtraction angiography (DSA) findings and the need to consider DCH in the differential diagnosis of multiple calvarial tumor-like lesions, we present this case and review the relevant literature regarding preoperative evaluation. The patient consented to the publication of their case, and the utmost confidentiality was maintained throughout the process.

## Case presentation

A 55-year-old female patient presented to our hospital in 2019 with a chief complaint of heaviness in the head. An MRI performed for intracranial evaluation revealed three tumor-like lesions near the SSS in the parietal and occipital regions (Figures 1A, 1B). The three tumor-like lesions measured 34×20×14 mm, 25×30×13 mm, and 10×12×8 mm from the front, in that order. As the patient exhibited no symptoms, and there was no bleeding within the lesions or surrounding cerebral edema, and in consideration of the patient's wishes, a policy of watchful waiting was adopted. At the MRI follow-up in 2025, the tumor-like lesions measured 36×22×14 mm, 26×30×13 mm, and 10×13×8 mm, with no enlargement or edema (Figures 1C, 1D). However, the patient expressed a desire for surgical intervention, resulting in a decision for craniotomy and resection. We compared the MRI images in this case with those of a parasagittal meningioma (Figures 2A-2F, 3A-3D, 4A-4D) [4,5]. The MRI scan revealed signs of hemosiderin deposition on T2-weighted images (T2WI) (Figure 2C). Contrast-enhanced T1-weighted imaging (T1WI) revealed findings suggestive of the dural tail sign (Figure 3B). DSA revealed no obvious feeding vessels (Figures 4A, 5A). Developmental venous anomalies (DVA) were observed in the vicinity of the tumor (Figure 5B), whereas distinct tumor enhancement was evident along the tumor margins in the meningioma (Figures 5C, 5D). In view of these findings, a meningioma was strongly suspected, and a craniotomy with tumor resection was planned.

**Figure 1:**
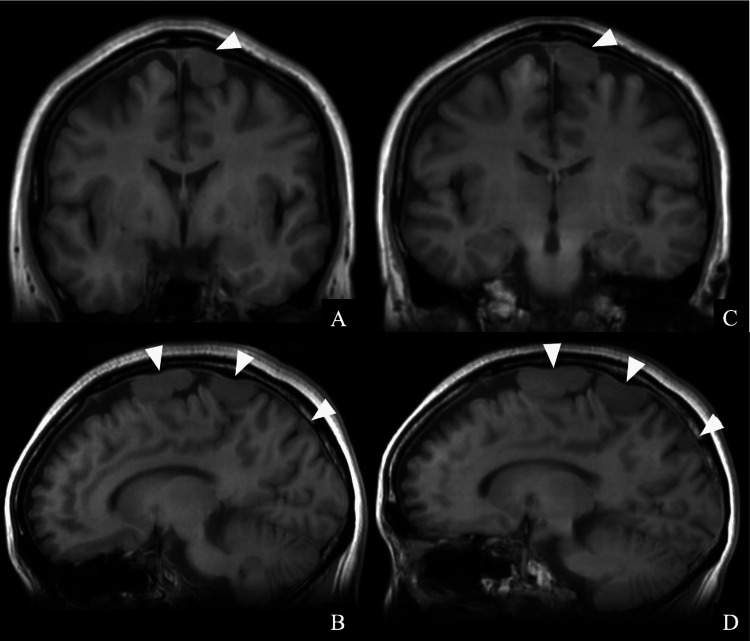
Coronal and sagittal T1-weighted MRI images obtained at the initial presentation and six years later. The MRI images (A, B) demonstrated three tumors in the parietal and occipital regions in proximity to the SSS (arrowheads). Following a period of six years, subsequent MRI in the coronal and sagittal planes (T1WI) (C and D) revealed no augmentation in the dimensions of the three lesions (arrowheads), and no manifestation of edema. SSS: superior sagittal sinus; T1WI: T1-weighted images

**Figure 2:**
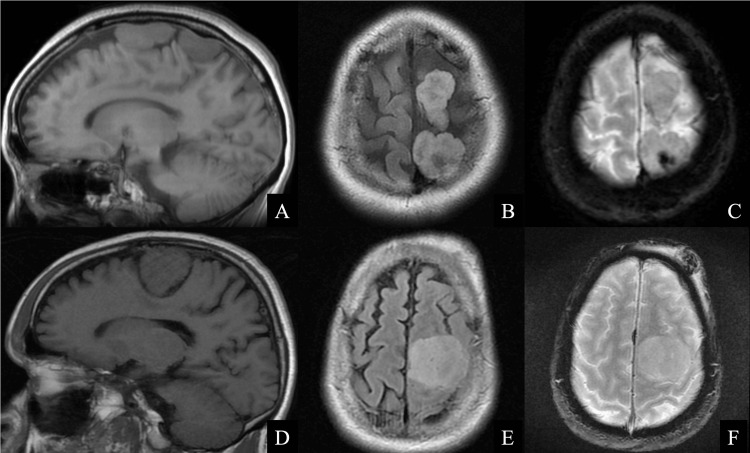
Structured MRI comparison of imaging features between the present case of dural cavernous hemangioma (DCH) and a parasagittal meningioma. The upper images (A, B, and C) show MRI scans of this case (DCH), while the lower images show scans of a parasagittal meningioma [4]. From left to right, the images are T1-weighted (A, D), FLAIR (B, E), and T2-weighted (C, F). On T1-weighted images, both lesions appear mildly hyperintense and well-defined. On FLAIR images, both lesions exhibit homogeneous hyperintensity throughout. The cavernous hemangioma (C) contains areas of hypointense signal on T2-weighted images (T2WI), suggestive of hemosiderin deposition. In contrast, the meningioma (F) shows no areas of low signal intensity within the tumorous lesion. DCH: dural cavernous hemangioma; FLAIR: fluid-attenuated inversion recovery

**Figure 3:**
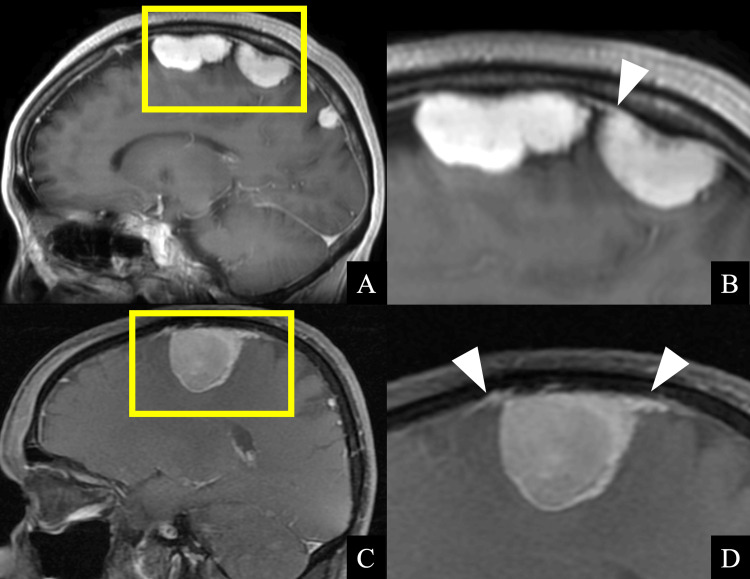
A comparison of contrast-enhanced MRI images of this case (DCH) and of a parasagittal meningioma, along with the findings of the dural tail sign. The upper row (A-C) shows contrast-enhanced T1-weighted MRI images of the present case of dural cavernous hemangioma (DCH), whereas the lower row shows images of a parasagittal meningioma acquired using the same sequence [4]. The regions outlined by the yellow frames in the original images are shown at higher magnification in panels B and D. Contrast enhancement is clearly visible in both lesions; however, the dural tail sign (arrowheads in B and D) is more pronounced in the meningioma than in the present DCH case. DCH: dural cavernous hemangioma

**Figure 4:**
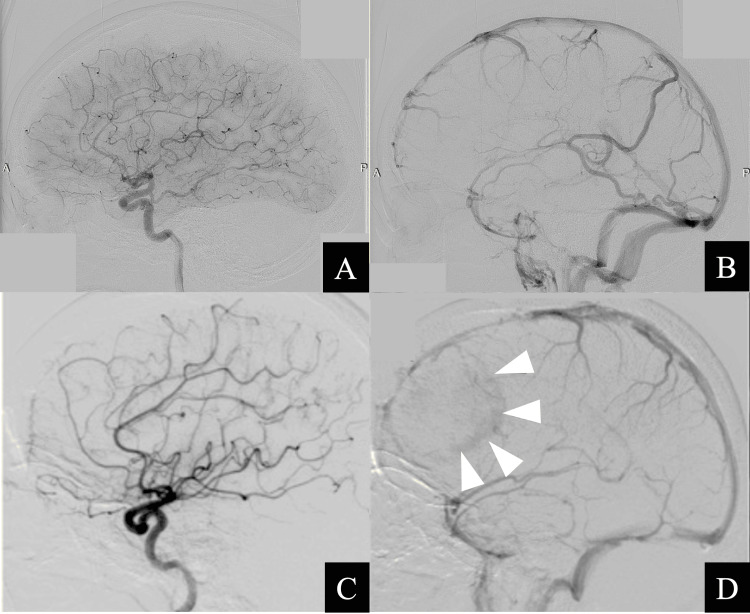
A comparison of internal carotid artery angiography (ICAG) images of this case (DCH) with those of a parasagittal meningioma. The images (A and B) in the upper row are ICAG scans of this case (DCH), while those in the bottom row (C and D) are ICAG scans of a parasagittal meningioma [5]. The images on the left (A and C) show the arterial phase, and the images on the right (B and D) show the venous phase. No tumor enhancement is observed in this case (DCH), whereas distinct tumor enhancement is evident along the contours of the tumor (arrowheads) in the meningioma. DCH: dural cavernous hemangioma

**Figure 5:**
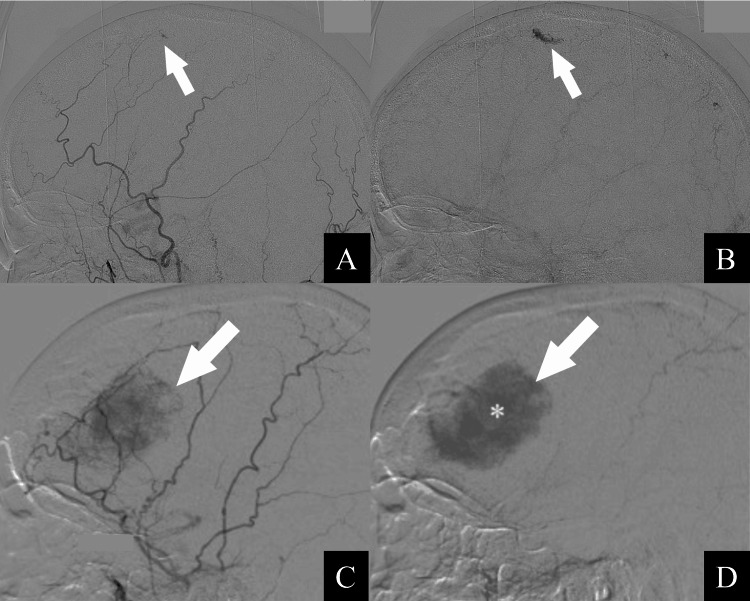
A comparison of external carotid artery angiography (ECAG) images of this case (DCH) with those of a parasagittal meningioma. Images A and B are ECAG scans of the present DCH case, whereas C and D show ECAG scans of a parasagittal meningioma [5]. Images A and C represent the arterial phase, while B and D represent the venous phase. No tumor enhancement is observed in the DCH case (A and B); however, developmental venous anomalies are visible (arrowheads in A and B). In contrast, distinct tumor enhancement (asterisk in D) is observed along the tumor margin (arrowheads in C and D) in the meningioma. DCH: dural cavernous hemangioma

Tumor resection

In the supine position, under general anesthesia, the surgical plan was to resect two lesions on the parietal side. These were considered to exert relatively stronger compression on the brain parenchyma than the other one tumor lesion. The craniotomy field was delineated to encompass the two tumor lesions. A skin incision was made circumferentially around the craniotomy field, with the base located on the mid-skull base. The temporalis muscle was preserved on the flap side, and the flap was retracted. The following five burr holes were drilled: three directly above the SSS and two laterally to it. The dura mater was retracted in a U-shape, with the SSS side acting as the base (Figure 6A). Microscopic dissection was then undertaken, leaving the portion near the SSS firmly adherent to the dura mater from the ventral tumorous lesion, while advancing the separation of the areas adherent to the brain parenchyma. The tumorous lesion was decompressed internally using ultrasonic surgery, with the appropriate caution exercised to avoid the compressed bridging veins identified on DSA (Figure 6B). Despite meticulous examination of the surrounding inflow vessels, no discernible vascular structures supplying the tumorous lesion were identified. Despite the presence of vascular clustering within the lesion, it did not demonstrate an inherent tendency to bleed. The specimen exhibited a mulberry-like appearance, characterized by a dark purple hue and a relatively firm texture. Internal observation revealed the presence of numerous vascular structures (Figure 6C). The dissection was performed circumferentially, incorporating the bridging vein, while meticulously preserving the area suspected to be the dura mater attachment site, with the objective of averting SSS injury. The subsequent transection and resection of the area in proximity to the SSS was undertaken, with meticulous attention paid to the dura mater attachment. The tumor resection margin was meticulously cauterized to prevent SSS occlusion. The second lesion was meticulously dissected and transected, with the utmost care taken to preserve the integrity of the bridging vein. Following confirmation of adequate hemostasis, the microsurgical procedure was completed (Figure 6D). The dura mater was primarily reconstructed, the autologous bone was replaced, and the layers were sutured to conclude the surgery.

**Figure 6:**
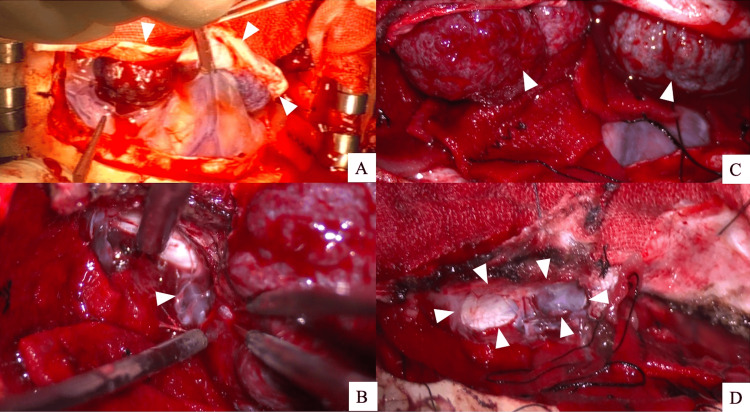
Intraoperative images showing tumor resection. Intraoperative images are presented for further elucidation. The dura (arrowheads) was inverted in a U-shape at the base on the SSS side (A). The bridging vein (arrowheads) was compressed by the tumor and was meticulously dissected from the ventral tumor (B). The tumor (illustrated by arrowheads) manifested as diminutive mulberries, exhibiting a dark purple hue, a relatively high degree of hardness, and an abundance of vascular structures (C). The tumor was resected by meticulously dissecting the areas in proximity to the SSS (arrowheads) and the dural attachment site, while ensuring the preservation of the SSS (D). SSS: superior sagittal sinus

Postoperative course

Contrast-enhanced MRI performed on the day after surgery revealed no evidence of residual lesions, brain parenchymal contusions, or postoperative hemorrhages (Figures 7A, 7B). The postoperative pathological diagnosis of the resected lesion was DCH. Histological analysis revealed the presence of clusters of vascular structures characterized by dilated lumens, which appeared to be continuous with the dura mater. The overall appearance was characterized by a cavernous quality, with a conspicuous absence of distinct arterial or venous wall structures. Furthermore, no evidence of cellular proliferation, suggestive of a meningioma, was observed. The absence of fragile wall structures within the brain parenchyma is notable, and the histological similarity to CH arising in soft tissues of meningeal origin is evident (Figures 7C, 7D). Given the location, perivascular hemangioma was also suspected, and a re-examination was performed, but the diagnosis remained unchanged. Immunostaining revealed that the inner surface of the spongy lesion is lined with CD31- and CD34-positive vascular endothelial cells, supporting the diagnosis of DCH. No proliferation of EMA-positive meningothelial cells was observed within the lesion, and there was no positive reaction to STAT6. Additionally, a small area of EMA-positive tissue was observed primarily adjacent to the lesion. However, upon examination of the HE specimen, it was found to consist of fibrous tissue accompanied by Rosenthal fibers and was judged to be glial tissue. The MIB-1 (Ki-67) index was 1% or less. A subsequent MRI re-examination on day seven revealed no significant abnormalities, and the patient was discharged from the hospital. A subsequent MRI scan performed one month after discharge revealed no enlargement of the remaining lesion and no postoperative complications. The residual lesion in the more dorsal region is being monitored and has not increased in size since it was first observed. No surgical treatment is being undertaken.

**Figure 7:**
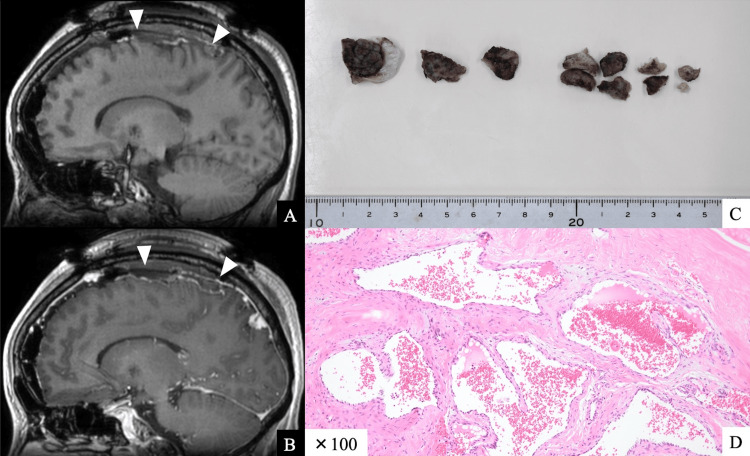
Postoperative MRI and pathological examination. On the day following the surgical procedure, plain T1WI (A) and contrast-enhanced T1WI (B) scans revealed no residual lesions, contusions, or posthemorrhagic changes in the frontal or parietal lobes (arrowheads). A subsequent pathological examination of the tumors post-surgery revealed a diagnosis of DCH. Histological analysis revealed clusters of vascular structures characterized by dilated lumens extending from the dura. The specimen manifested a spongy appearance, devoid of arterial or venous wall structures, and exhibited no cellular proliferation indicative of a meningioma (C, D). T1WI: T1-weighted images

## Discussion

A review of recent case reports of DCH reveals that fewer than 30 cases of dome-region DCH have been documented [2,3]. Reports of multiple occurrences in the dome region are uncommon. CHs within the brain parenchyma are known to exhibit a popcorn-like internal structure with mixed low- to high-signal intensity, showing a multi-phase hematoma on T2WI [6]. Furthermore, the presence of hemosiderin deposition within the lesion and at its margins is demonstrated on T2WI [7]. On DSA, feeding vessels are frequently not visible, and the presence of a DVA is occasionally documented. The enhancement of contrast is subject to variation, ranging from minimal to marked [8]. Conversely, parasagittal meningiomas characteristically manifest as relatively well-defined enhancement on contrast-enhanced T1WI, with a discernible dural attachment site frequently exhibiting the dural tail sign. Frequently, contrast-enhanced angiography reveals feeding vessels. In this particular instance, T2WI revealed an internally heterogeneous, well-defined mass lesion with minimal edema. T2WI revealed a part of the hemosiderin deposition around or within the lesion. Contrast-enhanced T1WI confirmed the presence of a DVA, but it revealed findings that were suggestive of a dural tail sign. This finding indicated continuity with the adjacent dura mater. In view of the aforementioned data, the hypothesis that the tumor-like lesion in this case is a meningioma of the dome is highly probable. However, the absence of clear feeding vessels on DSA made it difficult to actively support a meningioma diagnosis. Moreover, a review article by Hassanzadeh et al., which summarized recent case reports, also failed to identify any cases of DCH with feeding vessels visible on DSA [3]. Consequently, DCH should be incorporated into the differential diagnosis at the preoperative stage in this particular case. As posited in a Japanese case report by Yonezawa et al., DCH is frequently characterized by high signal intensity on T2WI and is often devoid of the dural tail sign on contrast-enhanced T1WI [9]. The high signal intensity observed on T2WI is regarded as being relatively specific and an important distinguishing feature. Furthermore, cerebral angiography frequently reveals the presence of inflow vessels from the external carotid artery system in meningiomas, while cavernous hemangiomas may demonstrate an absence of inflow vessels or inflow vessels from the internal carotid artery system. In the reported case, the lesion exhibited a faint low signal intensity on T1WI, high signal intensity on T2WI and fluid-attenuated inversion recovery (FLAIR) sequences, and slightly heterogeneous enhancement with contrast. The margins of the lesion were found to be slightly irregular, with partial enhancement of the surrounding dura mater, suggesting a dural tail sign. No peritumoral edema was observed. Cerebral angiography revealed a faint tumor shadow in the late arterial phase, with no obvious feeding vessels identified.

It is generally accepted that DSA is beneficial in the preoperative evaluation of meningiomas and for embolization purposes, as it helps to mitigate the risks associated with surgical intervention. Conversely, since parasagittal meningiomas are relatively superficial lesions, the identification of feeding vessels is considered technically less challenging during surgery. Advancements in diagnostic techniques for non-invasive imaging have led to a paradigm shift in clinical practice, in which some institutions now perform tumor resection based exclusively on MRI findings, eschewing the conventional DSA procedure. However, in the context of preoperative diagnosis of meningiomas, although DSA is considered more invasive than CT and MRI, it is considered to have the highest sensitivity and specificity for assessing the structure of peritumoral vessels [10]. Consequently, when contemplating surgical intervention for parasagittal tumors, the implementation of preoperative DSA is advantageous, irrespective of the definitive diagnosis being meningioma or DCH. This preoperative evaluation provides a highly reliable assessment of the vascularity. Moreover, in the course of evaluating asymptomatic parasagittal tumors, if DSA diagnoses a DCH, the treatment option of limiting the procedure to a biopsy or observation without surgery can be proposed. In this particular instance, preoperative DSA revealed findings consistent with DCH as a differential diagnosis, a diagnosis that was subsequently confirmed by the postoperative definitive diagnosis. Consequently, implementing DSA as a preoperative evaluation is advantageous not only for mitigating surgical risks but also for informing future treatment strategies, thereby outweighing the procedural risks. Therefore, if a parasagittal meningioma is suspected and the resection is deemed to be relatively straightforward, carrying out a DSA as a preoperative examination may enable the procedure to be less invasive, for instance, by limiting the intervention to a biopsy.

## Conclusions

This study details a patient presenting with multiple DCHs. In this case, differential diagnosis from a parasagittal meningioma proved extremely difficult, and craniotomy and resection were ultimately performed. Since preoperative DSA identified DCH as a differential diagnosis in addition to parasagittal meningioma, it was suggested that this modality is useful in determining the treatment strategy. It is clear that further research is needed to establish the most effective method for the preoperative diagnosis of DCH.
